# Detection of Dementia-Related Abnormal Behaviour Using Recursive Auto-Encoders

**DOI:** 10.3390/s21010260

**Published:** 2021-01-02

**Authors:** Damla Arifoglu, Yan Wang, Abdelhamid Bouchachia

**Affiliations:** 1 Department of Computer Science, University College London, London WC1E 6BT, UK; d.arifoglu@ucl.ac.uk; 2 School of Electronic and Information, Zhongyuan University of Technology, Zhengzhou 450007, China; 3 Department of Computing, Bournemouth University, Poole BH12 5BB, UK; abouchachia@bournemouth.ac.uk

**Keywords:** activity recognition, cognitive impairment, abnormal behaviour detection, hierarchical learning, recursive auto-encoders, data generation

## Abstract

Age-related health issues have been increasing with the rise of life expectancy all over the world. One of these problems is cognitive impairment, which causes elderly people to have problems performing their daily activities. Detection of cognitive impairment at an early stage would enable medical doctors to deepen diagnosis and follow-up on patient status. Recent studies show that daily activities can be used to assess the cognitive status of elderly people. Additionally, the intrinsic structure of activities and the relationships between their sub-activities are important clues for capturing the cognitive abilities of seniors. Existing methods perceive each activity as a stand-alone unit while ignoring their inner structural relationships. This study investigates such relationships by modelling activities hierarchically from their sub-activities, with the overall goal of detecting abnormal activities linked to cognitive impairment. For this purpose, recursive auto-encoders (RAE) and their linear vs. greedy and supervised vs. semi-supervised variants are adopted to model the activities. Then, abnormal activities are systematically detected using RAE’s reconstruction error. Moreover, to apply RAEs for this problem, we introduce a new sensor representation called raw sensor measurement (RSM) that captures the intrinsic structure of activities, such as the frequency and the order of sensor activations. As real-world data are not accessible, we generated data by simulating abnormal behaviour, which reflects on cognitive impairment. Extensive experiments show that RAEs can be used as a decision-supporting tool, especially when the training set is not labelled to detect early indicators of dementia.

## 1. Introduction

Studies indicate that 17% of the population is aged over 65 in the UK and one million people will have dementia by 2025, and this will increase to two million by 2050 [1,2,3]. These numbers underline a situation which presents a certain level of criticality that needs to be managed. Cognitive impairment is a condition-affecting the memory and thinking abilities of elderly people [4]. This situation makes the elderly people dependent on their caregivers. However, studies show that age-in-place can help to mitigate the affects of cognitive decline. Providing a living environment in a smart home can assist elderly people suffering from dementia to lead an independent life. Moreover, tracking daily activities of elderly people at such a smart home would be helpful to detect the early indicators of dementia.

The indicators of cognitive impairment can be observed in daily activities, such as cooking and eating [5,6]. Monitoring the trends over time and tracking the changes in activity patterns, such as getting up repeatedly during the night and failure to complete tasks, would be useful to understand the markers of cognitive decline. For example, an elderly person suffering from Alzheimer’s may have abnormalities in their sleeping patterns, such as waking up or going to the toilet in the middle of the night. Moreover, there can be an abnormality in their eating habits (forgetting to have dinner, for example), or they may suffer from the consequences of dehydration because of forgetting to drink water. They may also get confused and make mistakes while performing activities such as running the dishwasher; they may confuse names on a phone book; they might forget to turn off heaters and kitchen utilities [7].

In-home automatic assessment of cognitive decline has been the subject of many studies [8,9,10,11,12]. Currently, questionnaires or in-person examinations are being used by experts to evaluate the cognitive status of elderly people. The work reported in [11] was a comparison of the paper-based Montreal Cognitive Assessment and its electronic version (eMoCA) from 401 participants in two groups. A demographic questionnaire was built into the eMoCA. The study presents that the eMoCA provides the potential to screen for early changes in cognitive function and the access to rural or remote communities. In [12], the authors assumed that leisure-time physical activity (LTPA) is protective against decline in cognitive performance. They evaluated the cognition assessment using the in-person questionnaires to the participants. Study results show the independent association between a low level LTPA and a greater decline in cognitive performance. However, examination methods of this kind poorly represent the cognitive status of an elderly person, since they only depend on pre-defined questions asked in a given short time. Our study relies on the idea that indicators of cognitive impairment can be observed in daily activities. Thus, monitoring the activities of an elderly person in a smart assisted environment would be helpful to assess the cognitive status. This system could be used as a decision-supporting system for caregivers and medical doctors to take action towards improving their life quality.

Daily activities are often composed of several sub-activities [13]. For example; the activity “preparing coffee” consists of the following sub-activities: boiling water; taking a cup; mixing coffee and water. These sub-activities are important in the detection of abnormal behaviour related to dementia. For example, an elderly person suffering from cognitive decline may get confused during the performance of an activity and this may result in repetition or skip of some sub-activities. The repetition frequencies of sub-activities and their correlations can be clues regarding the abnormal behaviour arising from cognitive impairment. Unfortunately, existing studies treat each activity as an atomic unit and fail to model activities based on their sub-activities, and thus fail to capture the relationships among sub-activities, which might be important in the context of dementia. This study addresses this shortcoming by constructing activity instances hierarchically from their sub-activities. Activity recognition resembles scene parsing or phrase detection, which are hierarchical learning problems. Inspired by solutions to these problems [14,15,16], we explored recursive auto-encoders to model daily activities from their low-level sub-activity structures hierarchically and then detect abnormal behaviour arising from cognitive decline.

Unfortunately, there exists no publicly available dataset on abnormal behaviour of people with dementia. Producing such a dataset would require time and an adequate experimental environment. When there is no real-world dataset available, data simulation can be a solution [17,18,19,20]. Given the scarcity of such data, simulating daily life abnormal behaviours of elderly people suffering from dementia would be helpful for providing automatic assessment methods. Thus, in this paper, a method is proposed to artificially produce abnormal activities reflecting on typical behaviour of elderly people with dementia.

**Figure 1:**
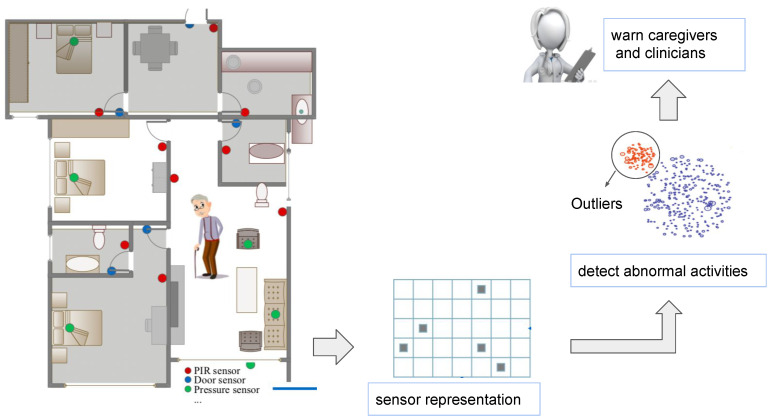
Overview of the proposed method.

The proposed application would be used as a cognitive status assessment method in the natural flow of daily life of elderly people suffering from cognitive decline. The proposed method would be used as a warning and decision supporting system rather than a decision making system. As described in Figure 1, the model learns the normal activities and detects the possible candidates for abnormal behaviour, which are actually deviating sensor representations from the normal ones. Then the detected abnormal activities are presented to the caregiver or the medical doctors to support their decision making. Final decisions would be given by the clinicians.

The contributions of this paper are three-fold.

- Simulation of abnormal behaviour: A method is presented to simulate abnormal behaviour stemming from cognitive decline. More specifically, activity-related (repetition of activities and sleep disorder anomalies) and sub-activity (confusion)-related abnormal behaviour instances are generated from real-world data.
- A new sensor representation: Raw sensor measurements coming from sequential data are represented using raw sensor triggering information rather than a bag-of-words style approach. This representation encodes granular-level information such as the frequency of each sensor activation and their relative activation order.
- Modelling activities hierarchically: Recursive auto-encoders and their variants are used to model sensor-based daily activities based on their sub-activities and detect abnormal behaviour-related to cognitive impairment.

The rest of the paper is organised as follows. Section 2 summarises the literature work. Section 3 describes the dataset used and explains dementia-driven data generation; it also presents the sensor representations along with the variants of the auto-encoder models for abnormal behaviour detection. Section 4 presents the experimental settings and results. Section 5 provides a discussion of the results. Finally, Section 6 concludes the paper.

## 2. Related Work

Automatic assessment of cognitive impairment has been tackled using many machine learning approaches, such as support vector machines (SVMs), naïve Bayes (NB) methods [21], restricted Boltzmann machines (RBMs) [22], Markov logic networks [9,23], hidden Markov models (HMMs) [19,24], random forest methods [20], hidden conditional random fields [25], recurrent neural networks (RNNs), convolutional neural networks (CNNs) [26,27] and some hierarchical models [28,29].

Cognitive Assessment Studies: In [30,31], participants were asked to complete a pre-defined set of tasks and based on their performance, their cognitive status was evaluated. This score was calculated based on the duration of the activity and the sensor activations. In [32], the authors focused on kettle and fridge usage and sleep patterns. The cognitive status of a person was assessed based on the kettle and fridge usage times, durations and frequencies; and the duration of sleep. In [33], the authors designed games to assess the cognitive status of an elderly person. Unfortunately, these assessment methods were not performed in the natural flow of daily activities. Rule-based systems require trained experts and manually designed and integrated rules for each individual person since daily life habits change from person to person. For example, while one person has the habit of going to toilet frequently during the night, this routine might be abnormal for another person. On the other hand, our method does not involve expert input, because it learns the habits of people automatically from training data. In this study, we aim to detect abnormal behaviour in a real-life scenario and in the natural flow of daily living without providing any instructions, rules or tasks.

Deep Learning Studies: In [34], features were extracted and selected from sequential data using RBMs. In [35], convolutional neural networks (CNNs) and long short term (LSTM) recurrent neural networks (RNNs) were used to recognise activities from wearable sensors. In [36], the authors explored convolutional and recurrent approaches on movement data captured with wearable sensors. In [37], the authors utilised CNNs to classify activities using smartphone sensors. In [38], features from raw physiological signals were extracted using CNNs, and a multivariate Gaussian distribution was exploited to identify risks. Unlike our work, these studies exploited wearable sensor data, which would not be applicable for our task, since elderly people would be annoyed by wearing sensors. In [26,27], the authors exploited RNNs and CNNs to detect abnormal behaviour stemming from cognitive decline; however, these studies failed to capture the intrinsic structure of activities and cannot detect anomalies occurring at the sub-activity level.

Data Generation Studies: In [19], the authors modified a real-world dataset to synthesise health-related abnormal behaviours. Daily living activities such as sleeping and waking up were chosen, and abnormal behaviours such as frequent toilet visits, no exercise and sleeping without dinner were synthesised. In [20], more data were synthesised using HMMs based on real data collected. To increase the realism of data simulation, the authors modelled the sensor events by a combination of Markov chains and the Poisson distribution. However, in both [19,20], it was not mentioned in detail how the data synthesis was done. In [17], the authors modified a real-life dataset, converting the rooms into activities. The authors focused on walking and eating in conjunction with the sleeping activity, and samples of these activities were manually inserted.

Hierarchical Modelling Studies: In [25], the authors exploited HCRF to detect abnormal behaviour by considering sub-activity and their relations. First, activities were recognised by using HCRF; then a threshold based method was used to detect abnormal behaviours. However, they did not build activities from their sub-activities; they looked for anomalies in sub-activities manually. For example, for an anomaly occurring in sub-activity “forget to turn off the tap”, they checked the HCRF confidence value calculated for this sub-activity specifically. In [9], the authors detected anomalies by exploiting a Markov logic network, which uses rule-based reasoning and probabilistic reasoning. Unfortunately, these rules would need to be changed based on the home environment, sensors and habits of the elderly. In [23], these rules were learned automatically by using a formal rule induction method. In our study, the abnormal behaviour is defined in the context of sequences considering their relationships with before and after activities, similarly to [9,25]. In [28], recursive auto-encoders (RAE) were used to cope with the scarcity of data. The authors applied transfer learning when there was limited data available. They learnt “normal” behaviour in a source household, and then transfered the parameters of a RAE to another house (source) to detect abnormal behaviour of dementia sufferers. In [29], graph convolutional networks (GCNs) were exploited to build daily activities from their granular-level structures in order to detect abnormal behaviour arising from cognitive impairment.

Impressive results have been obtained with recursive models in hierarchical learning problems, such as parsing, sentence-level sentiment analysis and paraphrase detection and scene parsing [14,15,39]. In [40,41], auto-encoders were exploited for anomaly detection in time-series sequences. In [15], the authors used recursive auto-encoders for predicting sentiment distributions. Instead of using a bag-of-words model, hierarchical compositional semantics was exploited to understand the sentiment. Inspired by [14,15], we aim to hierarchically merge sensor readings coming from time-series sensor activation data. This model will be helpful to understand the intrinsic sub-structures of activities and to extract sub-activities.

Data Simulation Studies: Many studies used data simulations to cope with the scarcity of data [17,19,20]. In [28], transfer learning via recursive auto-encoders (RAE) was used to detect abnormal behaviour of elderly people when there was limited data available. First, normal behaviour was learned in a source household, and then the parameters of a RAE were transferred to another house (target) to detect abnormal behaviour. In [19], the authors modified a real-world dataset to synthesise health-related abnormal behaviour for their experiments. In [20], more data were synthesised using hidden Markov models (HMMs) based on a small set of real data collected. In [17], the authors modified a real-life dataset of an older adult converting basically the rooms into activities. In [28], recursive auto-encoders (RAE) via transfer learning were used to cope with the scarcity of data. First “normal” behaviour in a source household was learnt, and then the parameters of a RAE were transferred to another house (source) to detect abnormal behaviour of dementia sufferers.

Sensor Representations: The studies in the literature exploit binary, changing and lasting features [42]. However, these features were extracted from time-slice chunks within a given time and neglect the interaction between sensors, their triggering order and frequency. Similarly to our work, in [43], the authors tried to capture the relationship between the sensor activations. They learn an adjacency matrix reflecting the sensor topology in the house.

## 3. Materials and Methods

In this section, firstly, the dataset used is presented along with the simulation of the abnormal behaviour. Secondly, two different sensor representations, namely, bag-of-sensors and raw-sensor-measurement, are described. Thirdly, recursive auto-encoder models and their variants, namely, traditional and greedy RAEs are presented. Lastly, an abnormal behaviour detection method is summarised.

### 3.1. Dataset Description

The proposed RAE-based method was evaluated on Aruba testbed provided by CASAS smart home project [44]. In our study, we used three door and 31 motion sensors and excluded temperature sensors, since they do not add any additional information. The data were collected in 224 days and data were noted as sensor readings and time-stamps. In total, there are 11 daily activities, namely, “meal preparation”, “relaxing”, “eating”, “work”, “sleeping”, “washing dishes”, bed to toilet”, “entering home”, “leaving home”, “housekeeping” and “respirating” in this dataset. Unfortunately, Aruba dataset does not include any abnormal behaviour reflecting the cognitive status of elderly people with dementia. Therefore, we need to generate some artificial abnormal behaviour.

### 3.2. Simulation of Dementia-Related Abnormal Behaviour

In this study, we generate two types of abnormal behaviour observed in daily activities of elderly people with dementia: (i) activity and (ii) sub-activity-related abnormal behaviour. In activity-related anomalies, an activity itself is totally normal, but there is an anomaly related to its frequency or its occurrence time (before/after certain activities). On the other hand, a sub-activity-related anomaly occurs in the intrinsic structure of the activity (frequency of sensor activations, their order and correlation). In the first one, activities as a whole are repeated or forgotten; in the second one, some steps (sub-activities) of activities are forgotten or repeated.

#### 3.2.1. Activity-Related Abnormal Behaviour

An elderly person with cognitive decline has a tendency either to forget or repeat a certain activity [45,46]. This kind of abnormal behaviour is simulated by inserting certain activities within the sequence of the day. This simulation generates abnormal activities in an abnormal time of the day, such as cooking or going to the toilet in the middle of a night, showing degeneration of the sleep–waking cycle, which is a symptom of cognitive decline [45,47]. We injected the instances of the following activities into the normal activity sequences to generate abnormal activities related to the frequency: meal preparation, eating, work, washing dishes, leaving home and entering home. We injected relaxing, eating, bed to toilet and respirating into the normal activity sequences of sleeping activity to mimic abnormal behaviour stemming from sleeping disorders (see Algorithm 1). In total, we manually generate 77 abnormal activity instances.

**Algorithm 1:** Simulation of abnormal activities.

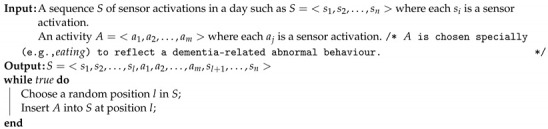



#### 3.2.2. Sub-Activity-Related Abnormal Behaviour

Elderly people with cognitive impairment may get confused during the performance of daily activities. As a result, they might tend to perform some sub-activities more than once, or change the orders of sub-activities within an activity. For example; during washing clothes activity, an elderly person may confuse how to use the washing machine, and may press the buttons of the machine a couple of times. Then the sensors on the machine would be triggered abnormally more than they should be.

**Figure 2:**
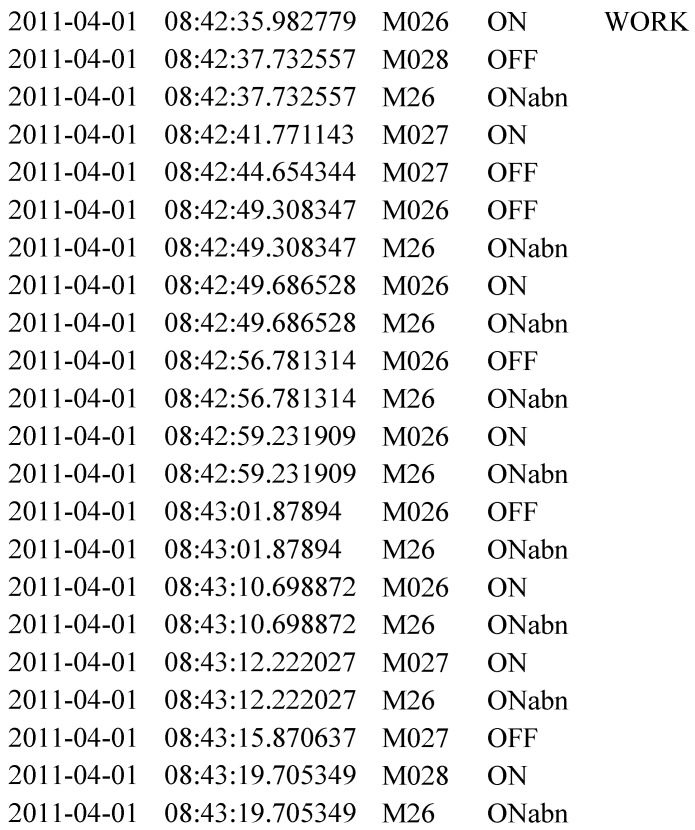
A snapshot for sub-activity-related anomaly synthesis. ONabn shows the inserted sensor activations.

The generation of this kind of anomalies is done by repeating some sensor activations in a given activity instance (see Algorithm 2). For this purpose, given random instances of working, eating, meal preparation and bed to toilet, we randomly repeat the sensors ($M_{26},M_{14},M_{18},M_{4}$ respectively) involved in these activities. Here, we can think that the sensor $M_{26}$ emulates the computer. Repeating the triggering of this sensor in working activity will emulate the confusion of using a computer. For example, assume that $S=s_{1},s_{2},s_{3},...,s_{n}$ is a randomly chosen sequence of working activity, where each $s_{i}$ represents a sensor activation. Here, the sensor $M_{26}$ is inserted at random locations with a random frequency. Then the modified *S* becomes $S=s_{1},M_{26},s_{2},s_{3},M_{26},...,M_{26},s_{n}$ which results in abnormal activations of that sensor (see Figure 2). In total 69 abnormal activities are generated in this category.

**Algorithm 2:** Simulation of abnormal sub-activities.

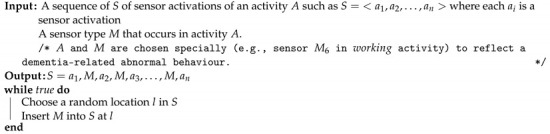



### 3.3. Feature Engineering

In this study, raw sensor readings are mapped onto two representations, namely, bag-of-sensors (BOS) and raw-sensor-measurement (RSM) representations.

#### 3.3.1. Bag-Of-Sensors (BOS)

This representation is the same as raw feature representation described in [42]. However, we name it BOS, since it resembles a bag-of-words representation in document recognition literature. This representation ignores the context of sensor events in a given duration. Firstly, time-slice chunks are segmented from raw sensor data using a sliding window approach [42]. A time-slice chunk can be thought of as a bag that collects the sensors which are triggered in a given time. A vector of length *N*, where *N* is the total number of sensors in the dataset, is initialised to zeros, and the sensors triggered at a given time are set to 1. This feature ignores the frequency and the order of activations.

**Figure 3:**
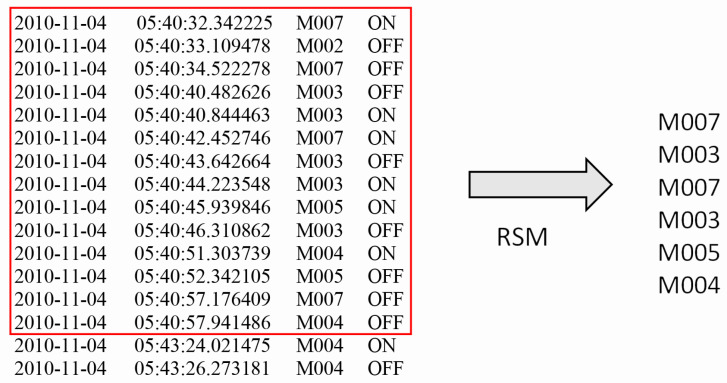
Raw sensor data and its RSM feature.

For example, sensor readings from Aruba dataset within a 1 minute time are shown in Figure 3. There are 34 sensors in Aruba test-bed. Thus BOS representation for this chunk will be 0011101000000000000000000000000000, where only the positions at 3,4,5,7 are set to 1. Although M003 is triggered two times and M005 is triggered only once, they have the same affect on the representation. Moreover, first M007 is activated and then M003 and so on; nevertheless, this order is lost in this representation.

#### 3.3.2. Raw-Sensor-Measurement (RSM)

In this version, the frequency and the correlation between the sensor activations are preserved. For example, given the one-minute data in Figure 3, the RSM representation will be $M_{7},M_{3},M_{7},M_{3},M_{5},M_{4}$. The representation is then mapped onto a one-hot encoded representation for each sensor activation. The extracted representation will be the variable size of (number of sensor activations in a given time window × number of total sensors in the dataset; in this example, 6×34), whereas BOS has a fixed size of (1 × number of total sensors in the dataset; 1×34 in this case). BOS feature ignores the relative order and the frequency of sensor activations, whereas this information is captured by the RSM representation. However, the order of sensor activations, their correlation with other sensors and their frequency are granular, important details to detect anomalies-related to dementia.

### 3.4. Auto-Encoder Models for Abnormal Behaviour Detection

An auto-encoder network is an architecture that takes an input and is trained to reproduce that input in its prediction layer. Auto-encoders are unsupervised since they do not need explicit labels during training. However, they work in a self-supervised fashion since they learn model parameters relying on training data. An auto-encoder consists of an encoder compressing the input, a decoder reconstructing the input and a loss function calculating the error between the real input and the reconstructed input.

In a recursive auto-encoder (RAE), which originated from [48], given two children, an encoding function first constructs the parent. Then the children are reconstructed by decoding function to calculate the loss. The same encoder and decoder are used at all levels of the tree recursively. We will be focusing on two types of RAEs—traditional RAEs and greedy RAEs.

#### 3.4.1. Traditional Linear Recursive Auto-Encoders

**Figure 4:**
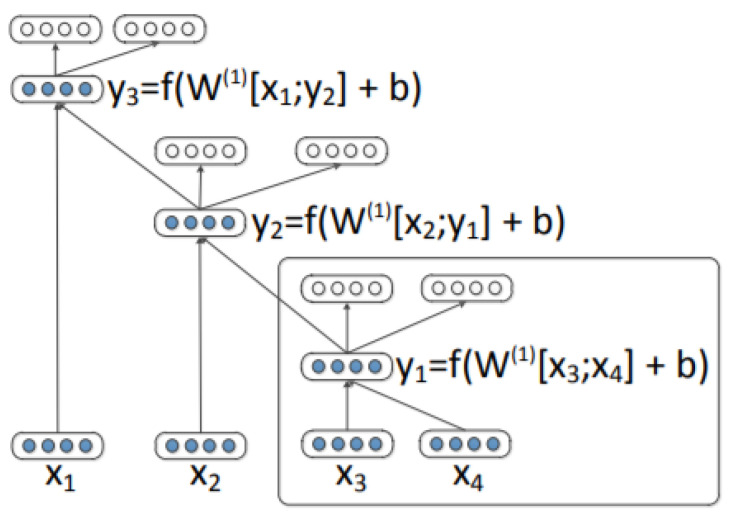
A recursive auto-encoder.

In a traditional RAE, a parent is constructed by merging a child with its neighbour. In Figure 4 (figure retrieved from [15]), a list of inputs $x=(x_{1},x_{2},x_{3},x_{4})$ is given. First, the children (c1,c2)=(x3,x4) are merged to calculate parent $y_{1}$ so that $p=f(W^{\left(1\right)}\left[c1;c2\right]+b^{\left(1\right)})$ where a weight matrix *W* is multiplied with the children. Then a bias term is added before applying an element-wise activation function such as tanh. Next, parent vector $y_{1}$ is merged with the next child $x_{2}$. The same procedure is applied recursively in all levels of the tree. Then, the model reconstructs the children in a reconstruction layer: $\left[c_{1};c_{2}\right]=g\left(W^{\left(2\right)}p+b^{\left(2\right)}\right)$. In the end, the reconstruction errors are minimised in a training phase to learn the model parameters. The reconstruction error is calculated as $E=P_{orig}\left(\left[c1;c2\right]\right)-P_{rec}\left(\left[c1;c2\right]\right)$. The process repeats until the full tree is constructed and a reconstruction error is obtained at each non-terminal node. The encoding and decoding weight matrices are learned by applying the back-propagation algorithm.

#### 3.4.2. Greedy Recursive Auto-Encoder

**Figure 5:**
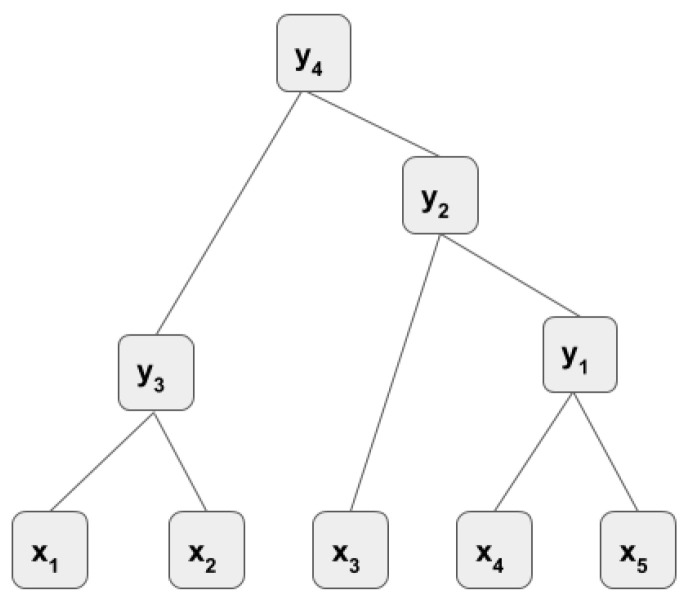
A greedy recursive auto-encoder.

In greedy RAE, two children that give the least reconstruction error are merged at each tree level. This greedy approach is described as follows. Assume that a sequence of instances $x_{1},x_{2},x_{3},x_{4},x_{5}$ is given (see Figure 5). First, the parent $p=x_{1},x_{2}$ of children [$x_{1}$,$x_{2}$] is encoded, then the children are reconstructed. The reconstruction error $e_{1}$ is calculated and kept in memory. Then, the merging is shifted to the right child where the parent of children [$x_{2}$, $x_{3}$] is encoded and the reconstruction error is calculated as $e_{2}$. This shifting is done until the last child is used. The minimum error among the errors $e_{1},e_{2},e_{3},e_{4}$ is chosen and the corresponding children are merged at that level. Let’s assume $e_{4}$ is the minimum, which is a result of merging of children [$x_{4}$, $x_{5}$]. The first merging for the first level of the tree is done as $y_{1}=x_{4},x_{5}$ and these children are represented by $y_{1}$. Then the merging for the second level is done with $x_{1},x_{2},x_{3},y_{1}$ and it continues in the same greedy manner until only one parent ($y_{4}$) remains in the last layer.

### 3.5. Abnormal Behaviour Detection

First, the dataset is divided into training and testing sets and the training set is used to learn the parameters for encoding function ($W^{\left(1\right)}$ and $b^{\left(1\right)}$) and decoding function $W^{\left(2\right)}$ and $b^{\left(2\right)}$ for a RAE model. Then test instances are given to constructed RAE trees to construct their parents. The main motivation behind is that given a training activity set of normal behavior, RAE learns a feature representation that encodes and models normal behaviour. Abnormal behavior is defined as the ones deviating from the expected behaviour. When a new test instance is introduced, the model will reconstruct the children with a small error, while the abnormal instances will be poorly reconstructed. Thus, the reconstruction error will be exploited to decide if an instance is normal or abnormal behaviour. Two different methods are used to construct RAE trees as follows.

#### 3.5.1. BOS Feature Merging Method

A sliding window of one minute is applied on the raw data (in both training and testing dataset) and sensor readings in each one window are mapped onto BOS representation (Section 3.3). Then a window size of *w* is used to extract chunks from these BOS representations. Thus, these chunks have a size w×n, where *n* is the number of features (=34). Then each row of a chunk is merged with its next row using traditional RAE until only one parent is constructed in the end.

In Equation (1), the error between the original children $x_{1}$ and $x_{2}$, and their reconstructed versions $x_{1}^{\prime }$ and $x_{2}^{\prime }$ is calculated using the mean squared error (MSE). *N* is the total number of features that each $x_{i}$ has. Then the error of each parent is used to decide if there is an abnormality in children or not. Here, in a constructed RAE tree for an input, time-slices in 25 min chunks are spanned, and the relationship between each one-minute slice is taken into account during the mergings in RAE.

(1) $$E_{rec}\left(x_{1}\right)=1/N\sum_{i=1}^{N}{\left(x_{1i}^{{}^{\prime }}-x_{1i}\right)}^{2}$$

#### 3.5.2. RSM Feature Merging Method

First, each one minute time-slices are mapped onto RSM representation. Inspired by [15], where words in a sentence are merged by a RAE, we treat each sensor activation as a word and each extracted RSM as a sentence. For example, in the extracted RSM feature $M_{7},M_{3},M_{7},M_{3},M_{5},M_{4}$, each sensor activation, such as $M_{7}$, is treated as a word. Resembling a sentence, in a RSM representation the order of the words is important to decide the context of a sentence. The sensor activations in RSM representations are merged hierarchically by greedy RAE. Each sensor activation is represented as a one-hot encoding representation during the merging. Here, the error for each RSM tree is used to decide if that time-slice is abnormal or not. This error is decided in two ways. First, the average error of all parents in the tree is used. Second, the error of the last parent is used. The experiments with this feature is performed in two modes following the same procedure in [15].

Unsupervised RAE: In unsupervised RAE, activity labels are not used and RAE is trained as described in Section 3.4.1. Each sensor reading is represented by one-hot encoding and parents are constructed from the children. The error is calculated using MSE in Equation (4).

Semi-Supervised RAE: In semi-supervised RAE, the error at each parent node is a combination of unsupervised RAE error (see Section 3.4.1) and supervised error. Supervised error is calculated in the following way. Assume that we have the RSM input $x_{1},x_{2},x_{3},x_{4},x_{5}$, which is extracted within one minute duration from raw data (Figure 5). The activity occurred at that one minute, label *l* is used as the label for whole parents in the tree while the parents are used as the features. Each parent *p* can be seen as a feature describing the sub-tree under it. Then a softmax layer is added to each parent as follows.

(2) $$d(p;\theta )=softmax(W^{label}p)$$

where $\theta =(W^{\left(1\right)},b^{\left(1\right)},W^{\left(2\right)},b^{\left(2\right)},W^{\left(label\right)})$. Assume that there are *K* labels, $d_{k}\in {I\;R}^{K}$ is a K−dimensional multinomial distribution and $\sum_{k=1}^{}d_{k}$. Then softmax layer’s outputs can be used as conditional probabilities for a parent *p* as $d_{k}\left(p;\theta \right)=p\left(k|\left[c_{1};c_{2}\right]\right)$. Then, the cross-entropy (supervised) error is:

(3) $$E_{sup}\left(p,t;\theta \right)=-\sum_{k=1}^{K}t_{k}\log d_{k}\left(p;\theta \right)$$

where $t_{k}$ is the *k*th element of the multinomial target label distribution *t* for parent.

A weighted average of supervised error $E_{sup}\left(x_{1},x_{2}\right)$ and unsupervised error $E_{unsup}\left(x_{1},x_{2}\right)$ (Equation (1)) is used to calculate the final error (Equation (4)), where α is decided experimentally as a value between 0 and 1.

(4) $$E_{rec}\left(x_{1},x_{2}\right)=\alpha E_{unsup}\left(x_{1},x_{2}\right)+\left(1-\alpha \right)E_{sup}\left(x_{1},x_{2}\right)$$

## 4. Results

In this section, first, the experimental set-up is described summarising implementation details and model parameters; then the results with different sensor representations and the proposed RAE-based method are presented along with the compared methods. We also present the results showing the classifiers’ performance and show n-gram patterns for the extracted sub-activities.

### 4.1. Experimental Set-Up

First the dataset was divided into training (139 days) and testing sets (70 days), where 15 days were used for validation. The testing set was modified to include sub-activity and activity-related anomalies. The modifications (Section 3.1) were done separately, which resulted in two different testing sets. We analysed sub-activity and activity-related anomalies separately to see the affects of RAEs on both types of anomalies individually.

We compare our results of RAE with RNNs (long short term variants), CNNs, HMM, NB and CRF. For comparison experiments, BOS feature is used since we need a fixed-length feature representation for these experiments. These models are supervised and they classify instances based on a confidence value. The models assign a class label to each instance with a confidence value. Firstly, the means of confidence values of training instances for each class were calculated as in Equation 5. If the model recognised a test instance with a confidence value smaller than a threshold, that instance was flagged up as an abnormal activity.

(5) $$m_{j}=1/N\sum_{t=1}^{N}p_{t}$$

where $m_{j}$ is the mean confidence value of class *j* and $p_{t}$ is the confidence value for training instance *t* of that class and *N* is the total number of instances in that class.

Keras Deep Learning library’s [49], and Theano’s [50] Python implementations of the CNNs and LSTM were used in this study. Experiments with NB, HMM, HSMM and CRF were conducted on the implementation provided in [42], which was implemented in Matlab. In the CNN and RNN experiments, the Adam optimiser [51] was used and the instances were fed into the system with a batch size of 20. In CNN experiments, a time-series window of length 10 seconds was extracted from the raw sensor reading data based on a sliding window approach. The CNN model had the following layers: A 2D convolutional layer (with 20 kernels of size 5×10), a max pooling layer (with a pooling size of 2×2), a 2D convolutional layer (with 10 kernels of size 10×15), a max pooling layer (with a pooling size of 2×2) a flattened layer and two dense layers of size 128 and 50, followed by a softmax layer to do the classification. In LSTM two hidden layers of 50 and 100 nodes were used. Then, dense layers of size 100, 128 and 50 were added to the network, followed by a softmax layer. There were drop-out layers with a probability of 0.5 between every two layers in both CNN and LSTM models.

RAE experiments were performed in two ways. The first experiment was conducted with BOS feature and it was implemented on Theano and Python, and the second experiment was based on Socher et. al.’s Matlab implementation [15] and performed with the RSM feature.

Abnormal behaviour detection success was measured using true positive rate (TPR) and false positive rate (FPR) (see Equations (6) and (7)). TPR vs. FPR values for different thresholds are shown on a receiver operating characteristic (ROC) curve. Additionally, area under curve (AUC) was calculated for each model to interpret the results in a better way. True positive rate (TPR) gives us an idea about the correctly detected instances which are abnormal. FPR calculates the percentage of mislabelled normal instances—in other words, it reflects the method’s ability to differentiate between normal and abnormal behaviour.

(6) $$\begin{array}{cc} \mathrm{TPR}\;\left(\mathrm{Sensitivity}\right) & =\frac{TP}{\left(TP+FN\right)} \end{array}$$

(7) $$\begin{array}{cc} \mathrm{FPR}\;\left(\mathrm{Specificity}\right) & =\frac{TN}{\left(TN+FP\right)} \end{array}$$

Precision, recall, accuracy and F-measures, as depicted in Equations (8)–(10), are used to evaluate classifier performance. Here, TP is true positive, TT is total number of instances, TP is total true labels, TI is total of inferred labels, *N* is the number of classes in a specific class of the dataset and Total is the total number of instances of all classes in the dataset. For each class, precision and recall are calculated separately and then the average is taken over all classes. As our dataset is unbalanced, these measures give us a better idea about the success of the models used. In unbalanced datasets such as daily activity ones, some certain classes (such as going to toilet) appear more than others (such as leaving home). Thus, our measure takes the average precision and recall over all classes and considers the correct classification of each class equally important. Accuracy calculates the total percentage of correctly classified time-slices; thus, more frequently occurring classes have larger weights [42].

(8) $$\begin{array}{cc} \mathrm{Precision} & =\frac{1}{N}\sum_{i=1}^{N}\frac{TP_{i}}{TI_{i}} \end{array}$$

(9) $$\begin{array}{cc} \mathrm{Recall} & =\frac{1}{N}\sum_{i=1}^{N}\frac{TP_{i}}{TT_{i}} \end{array}$$

(10) $$\begin{array}{cc} F\text{-}\mathrm{measure} & =\frac{2\times \mathrm{Precision}\times \mathrm{Recall}}{\mathrm{Precision}+\mathrm{Recall}} \end{array}$$

(11) $$\begin{array}{cc} \mathrm{Accuracy} & =\frac{\sum_{i=1}^{N}TP_{i}}{\mathrm{Total}} \end{array}$$

### 4.2. Evaluation of Features and Models

**Figure 6:**
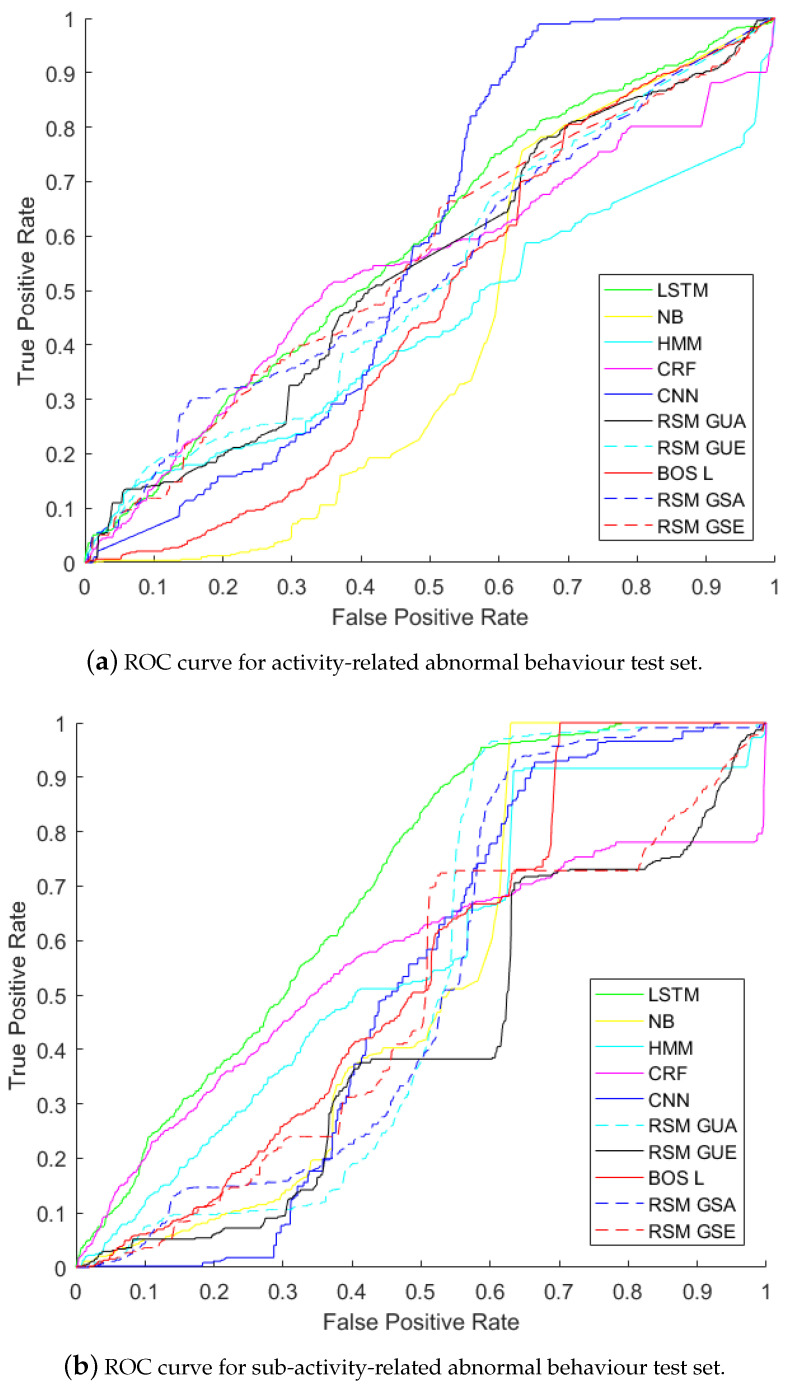
ROC curves for abnormal behaviour detection with both activity-related and sub-activity-related abnormal behaviours.

**Figure 7:**
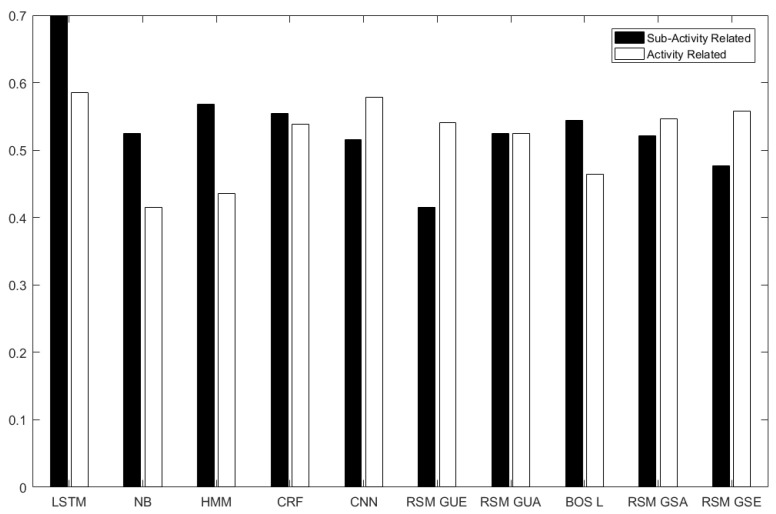
AUC histogram for the proposed methods and the comparison methods.

The results for anomaly detection are shown in Figure 6 and Figure 7. The abbreviations in Table 1 are used for the results.

Figure 6 and Figure 7 show the results on activity-related anomaly detection. The results show that LSTM is the best method giving the highest AUC (58.48%), and NB is the worst one (with AUC41.48%). Activity-related anomalies occur in the order of the activities involved, and LSTM is good at capturing temporal dependency between inputs, so it detects changes in the order of the activities. CNN comes as the second method (with AUC 57.79%). Instead of relying on given features, CNN extracts its own features taking spatial context into account. After CNN, RSM-GSE produced an AUC of 55.83%, which was followed by RSM-GSA (with AUC 54.59%) and RSM-GUE (with AUC 54.10%). Then CRF achieved an AUC of 53.80%. The next methods were RSM-GUA (with AUC 52.10%), BOS-L (46.45%) and HMM (AUC of 43.55%).

We see that RSM-GUE performs better than supervised methods NB, CRF and HMM. The reasons for this are as follows. HMMs are constrained to binary transition and emission feature functions, which force each instance to depend only on the current label and each label to depend only on the previous label. NB does not rely on any temporal dependency and it uses BOS measurement, neglecting both temporal context and granular-level details of each feature. Linear-chain CRF has limited memory, since it captures linear dependency between the current input and the previous one. RAEs learn hierarchical structures and the learned structures can capture more of the semantic relationships of sensor activations in RSM representation.

In Figure 6 and Figure 7, LSTM is the best method to detect anomalies related to sub-activities with an AUC of 69.91%. The second best method is HMM, and then comes CRF—AUCs of 56.76% and 55.43%. BOS-L achieved an AUC of54.36%. Please remember that BOS-L merged 25 instances of 1 min time-slices at each tree. Thus it could detect changes within 25 min and relate the changes between these time-slice instances. A sub-activity-related anomaly causes changes in the feature vector itself and in the neighbouring feature vectors. NB gives AUC of 51.20%, but it cannot capture temporal context. Greedy RAE with RSM model only takes 1 minute time-slices into account and constructs RAE trees, but unfortunately it cannot relate each RAE tree of 1 minute time-slices to the next time-slice, since it cannot take temporal information into account.

We see that RAE models do not give the best results when AUC values are compared. However, when an optimum threshold is chosen on the ROC curve, RAE models can perform as good as supervised methods. For example, in Figure 6a, RSM-GSE gives the same TPR (65%) and FPR (55%) with CNN and LSTM at the intersection point of their ROC curves. In Figure 6b, we see that RSM-GUA intersects with LSTM at TPR of 95% and FPR of 55%. AUC weights TRP and FPR equally. However, in some scenarios like ours, detection of TP is more important. For abnormality detection problem in skewed datasets, where the number of anomalies is much less than normal ones, TP is more important.

### 4.3. Classifier Performance

The next set of experiments was performed to evaluate the modelling ability of RAE and the representation ability of the reconstructed features of RAE. Even though the RSM representation has a variable length for each input, the RAE model outputs a fixed feature vector at the root node. The reconstructed representation of the root (size of 1×34) can be used as a final representation for the variable-length input and supervised classification methods can be trained with these features for further applications. We chose the J48 decision tree (the Weka implementation of the standard C4.5 algorithm) as our classifier due to its simplicity. The classifier results are depicted in Table 2. Firstly, classifier accuracy rates with BOS features are presented to provide a baseline for comparison. The classifier accuracy with BOS feature for activity-related test set is 81.37%, while it is 81.49% for sub-activity-related test set. The recognition accuracy rates with the RSM feature were as follows: 78.78% for activity-related anomaly test set; accuracy of 78.49% for sub-activity-related anomaly test set when supervised RAE was used; accuracy of 71.81% for activity-related anomaly test set; accuracy of 72.64% for sub-activity-related anomaly test set when unsupervised RAE was used.

Although an RSM representation gives less classification accuracy compared to BOS feature, it gives better precision and recall rates, which means experiments with RSM are good at providing class-specific detailed information and result in higher precision and recall rates. For example, the BOS-original experiment achieved a precision of 42.92%, recall of 42.31% and F-measure of 41.84% on the activity anomaly set, while RSM semi-supervised experiment achieved precision of 46.92%, recall of 47.23% and F-measure of 46.06%. For imbalanced datasets, RSM representation can be used where the accuracy is important, but also precision and recall on the least frequent classes are important. We see that supervised RAE calculates better features than unsupervised RAE and it gives very close classification accuracy rate with BOS feature, which shows that RSM has a high representation ability.

### 4.4. Pattern Extraction

**Figure 8:**
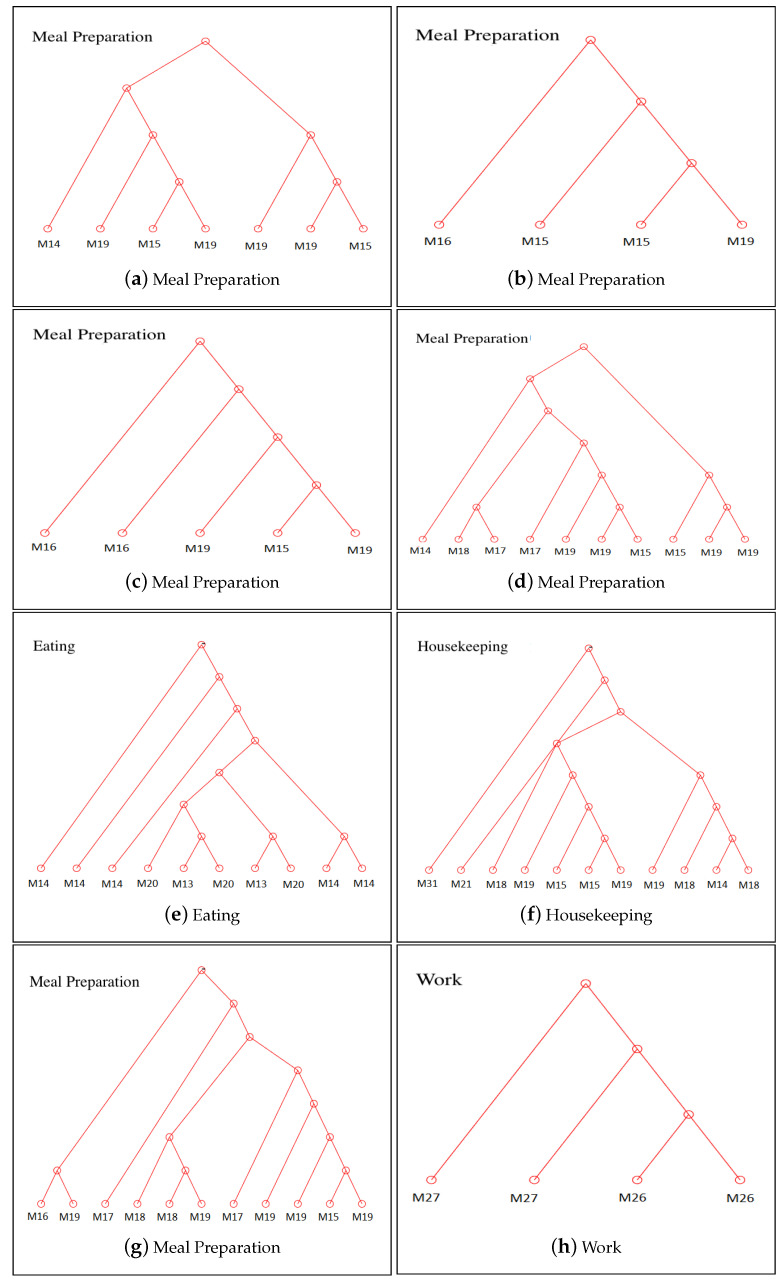
RAE trees constructed with the training set.

We also provide quantitative analysis to show how greedy RAE merges sub-activities in a hierarchical way to model activities. Sub-activities come together and form meaningful structures, which we call patterns. A sample set of constructed trees is shown in Figure 8. For example, we see that the sensors $M_{19}$ and $M_{15}$ are grouped together in the constructed trees for meal preparation activity. In Aruba testbed, these sensors are replaced close to each other, and when the resident performs meal preparation, these sensors are triggered one after another. Thus, they form a sub-activity pattern during the performance of this activity. The pattern constructed by these two sensors is identified as near the kitchen range and sink in [52], which supports our finding. Additionally, we see that the sensor $M_{16}$ is added to this sub-activity ($M_{15}$, $M_{19}$) which probably represents the cupboard usage during meal preparation. Another grouping of sensors, namely, $M_{17}$, $M_{15}$, $M_{19}$, shows another sub-pattern in this activity, which is again ($M_{17}$ and $M_{19}$) found as a movement pattern in [52]. We see that RAE hierarchically models these relations in the trees. In the eating activity, we see that $M_{13}$ and $M_{15}$ represent a sub-activity and $M_{14}$, $M_{13}$, $M_{15}$ represent another sub-activity, which is constructed by the sub-activity $M_{13}$, $M_{15}$ and the sensor $M_{14}$.

Moreover, we extract the most common and important patterns for each activity class in the following way. The idea is that the sensor readings, which are triggered one after each other frequently, represent a sub-activity (pattern). If they are seen together frequently in the training set, RAE learns to reconstruct them better and then in the test set; it gives less reconstruction error compared to the ones not seen frequently. We firstly sort all reconstruction errors of each node in the training set, and take the top 500 nodes with the least error. Then n-gram patterns are calculated with these top patterns. We calculate n-grams with only n=2 and n=3, which is already enough to see the patterns in the dataset. The n-grams are extracted from constructed the RAE trees by the supervised greedy method on the training set. The most frequent 2-grams and 3-grams are shown for each activity class in Table 3. For example, for the activity sleeping, the most frequent pattern is $M_{2}$, $M_{3}$; this makes sense because when we look at the sensor locations on Aruba testbed, we see that these sensors are on the bed and they will be triggered one after another during sleeping activity; thus they have a correlation. After extracting these frequent patterns (sub-activities), we can look for their errors in the RAE trees. If there is high error at those patterns, we can easily detect specific anomalies related to these patterns. For example, to check if the person is washing the dishes after a cooking activity, we can check sub-activity between the sink and the kitchen table and check the error of this sub-activity.

## 5. Discussion

Although LSTMs and CNNs outperform our proposed RAE-based method, one disadvantage of supervised models on our proposed method is that they require too much training data. Collecting and labelling that much training data is time consuming and a laborious task. Moreover, providing labelled data at the beginning would not be enough since observation of elderly people suffering from dementia in a smart home is a task which can be up to years. Thus, a continuous labelling of the data would be necessary. Moreover, activity classes need be fixed for supervised models. However, users tend to change their activity patterns in a time lapse of years. This would require the training set to be updated and labelled again. Thus, using RAEs to model activities is more advantageous than using supervised methods.

Moreover, although supervised methods give better AUC results than RAE models, they require labelling information which is tedious and time consuming task to obtain. In a case where getting a training set is difficult, RAE models can be an alternative to supervised methods. Moreover, detection of dementia indicators is a process spanning months and maybe years. In this time, the habits of residents may change and new activities may emerge. Thus, obtaining a training dataset and labelling it would not be sufficient since this labelling process would need to be repeated again when the activity labels change. However, with unsupervised methods such as RAE, no activity label data is used and the model can be updated at any time. Some of the supervised methods, such as CRF, take frequency information of each class instances into account and favours those classes in terms of classification. This would be a problem with imbalanced datasets like daily activity datasets, where abnormal detection of infrequent classes are important as well. However, RAE models do not learn class based parameters since they do not use class labels. We see that supervised methods used in the experiments tend to detect abnormal instances of frequent classes better than the others.

However, RAE models cannot relate one instance to another and neglects temporal information. Another problem with BOS is that it does not reflect the real status of an activity being performed. For example, people do not tend to close the room doors after they enter or leave. Once the door is open, the door sensor continues to emit 1. However, RMS feature representation only takes the activation of the sensor into account and then neglects the information that the door is left open. For scenarios where the door sensor is not important, it is good that the on status is not carried forward, but for abnormality detection scenarios such as leaving the door open, RMS feature would not be able to catch this information.

## 6. Conclusions

This paper proposed a method to detect abnormal behaviour associated with the cognitive impairment of elderly people. The abnormal behaviour of dementia sufferers is detected by decomposing activities into sub-activities to obtain a hierarchical tree structure. Using RAE, the promising results show that although this method cannot outperform methods such as LSTM, it can be used as an alternative when there is no training set or a limited training set available. Unfortunately, this method cannot relate one activity to another and neglects temporal information. We will extend the presented method in future investigations. Moreover, it is worth considering the off status of the sensors and investigating the effect on anomaly detection. The most important step in the future is to collect real-world data to study cognitive impairment. Our future work will be further evaluated with other popular semi-supervised or unsupervised models, such as semi-supervised ensemble isolation forest and local outlier factor, which are unsupervised anomaly detection methods. We will also investigate the applicability of deep unsupervised transfer learning or generative adversarial networks (GANs) for the detection of the indicators of dementia.

## Figures and Tables

**Table 1:** Abbreviations of the models used.

LSTM	Long Short Term Memory variants of RNNs
HMM	Hidden Markov Model
CRF	Conditional Random Field
CNN	Convolutional Neural Network
BOS-L	Unsupervised (U) traditional linear (L) RAE with BOS feature
RSM-GUA	Unsupervised (U) RAE with RSM and greedy (G) merging when the average (A) of all parent errors is used
RSM-GSA	Supervised (S) RAE with RSM feature and greedy merging when the average (A) of all parent errors is used
RSM-GUE	Unsupervised (U) RAE with RSM and greedy (G) merging when the error of root node (E) is used
RSM-GSE	Supervised (S) RAE with RSM and greedy (G) merging when the error of root (E) node is used

**Table 2:** J48 classifier precision, recall and accuracy rates (best results presented in bold font) with reconstructed features for both activity and sub-activity-related anomaly test sets.

**Model**	**Activity Anomaly Test Set**			
	**Precision**	**Recall**	**F-Measure**	**Accuracy**
**BOS—Original**	42.92%	42.31%	41.84%	81.37%
**RSM—Semi-supervised**	46.92%	47.23%	46.06%	78.78%
**RSM—Unsupervised**	47.33%	38.72%	37.89%	71.81%
**Model**	**Sub-Activity Anomaly Test Set**			
	**Precision**	**Recall**	**F-Measure**	**Accuracy**
**BOS—Original**	40.93%	42.08%	38.93%	81.49%
**RSM—Semi-supervised**	43.77%	42.77%	41.65%	78.49%
**RSM—Unsupervised**	42.84%	39.27%	37.89%	72.64%

**Table 3:** N-gram patterns extracted from training set.

Activity	2-Gram	3-Gram
Bed to Toilet	$M_{4}$, $M_{7}$ $M_{5}$, $M_{7}$	$M_{4}$, $M_{5}$, $M_{7}$ $M_{4}$, $M_{4}$, $M_{7}$
Meal Preparation	$M_{15}$, $M_{19}$ $M_{18}$, $M_{19}$ $M_{17}$, $M_{19}$	$M_{15}$, $M_{19}$, $M_{19}$ $M_{1}5$, $M_{18}$, $M_{19}$ $M_{15}$, $M_{16}$, $M_{19}$
Relaxing	$M_{6}$, $M_{9}$ $M_{8}$, $M_{9}$ $M_{9}$, $M_{13}$	$M_{9}$, $M_{9}$, $M_{13}$ $M_{9}$, $M_{9}$, $M_{10}$ $M_{9}$, $M_{13}$, $M_{20}$
Eating	$M_{8}$, $M_{14}$ $M_{6}$, $M_{14}$	$M_{9}$, $M_{14}$, $M_{14}$ $M_{10}$, $M_{14}$, $M_{14}$
Work	$M_{26}$, $M_{27}$ $M_{8}$, $M_{26}$	$M_{26}$, $M_{26}$, $M_{27}$ $M_{26}$, $M_{27}$, $M_{27}$
Sleeping	$M_{2}$, $M_{3}$ $M_{3}$, $M_{3}$ $M_{3}$, $M_{7}$	$M_{2}$, $M_{3}$, $M_{7}$ $M_{2}$, $M_{3}$, $M_{3}$ $M_{3}$, $M_{3}$, $M_{7}$
Washing Dishes	$M_{15}$, $M_{19}$ $M_{18}$, $M_{19}$ $M_{17}$, $M_{19}$	$M_{15}$, $M_{16}$, $M_{19}$ $M_{15}$, $M_{19}$, $M_{19}$ $M_{15}$, $M_{18}$, $M_{19}$
Housekeeping	$M_{14}$, $M_{20}$ $M_{13}$, $M_{20}$	$M_{15}$, $M_{18}$, $M_{19}$ $M_{14}$, $M_{18}$, $M_{20}$
Leaving Home	$M_{31}$, $D_{3}$ $M_{18}$, $M_{21}$	$M_{29}$, $M_{30}$, $D_{4}$ $M_{10}$, $M_{22}$, $m_{29}$
Entering Home	$M_{31}$, $D_{3}$ $M_{21}$, $M_{14}$	$M_{29}$, $M_{30}$, $D_{4}$ $M_{22}$, $M_{30}$, $D_{4}$
respirating	$M_{27}$, $M_{25}$	$M_{25}$, $M_{25}$, $M_{25}$
